# A QTL on mouse chromosome 12 for the genetic variance in free-running circadian period between inbred strains of mice

**DOI:** 10.1186/1740-3391-5-7

**Published:** 2007-10-31

**Authors:** John R Hofstetter, Doreen A Svihla-Jones, Aimee R Mayeda

**Affiliations:** 1 Department of Psychiatry, Richard L. Roudebush Veterans Administration Medical Center (VAMC), Indianapolis, IN 46202, USA

## Abstract

**Background:**

Many genes control circadian period in mice. Prior studies suggested a quantitative trait locus (QTL) on proximal mouse chromosome 12 for interstrain differences in circadian period. Since the B6.D2N*Ahr*^*d*^/J strain has DBA/2 alleles for a portion of proximal chromosome 12 introgressed onto its C57BL/6J background, we hypothesized that these mice would have a shorter circadian period than C57BL/6J mice.

**Methods:**

We compared circadian phenotypes of B6.D2N*Ahr*^*d*^/J and C57BL/6 mice: period of general locomotor activity in constant dark and rest/activity pattern in alternating light and dark. We genotyped the B6.D2N*Ahr*^*d*^/J mice to characterize the size of the genomic insert. To aid in identifying candidate quantitative trait genes we queried databases about the resident SNPs, whole brain gene expression in C57BL/6J versus DBA/2J mice, and circadian patterns of gene expression.

**Results:**

The B6.D2N*Ahr*^*d*^/J inbred mice have a shorter circadian period of locomotor activity than the C57BL/6J strain. Furthermore, the genomic insert is associated with another phenotype: the mean phase of activity minimum in the dark part of a light-dark lighting cycle. It was one hour later than in the background strain. The B6.D2N*Ahr*^*d*^/J mice have a DBA/2J genomic insert spanning 35.4 to 41.0 megabase pairs on Chromosome 12. The insert contains 15 genes and 12 predicted genes. In this region *Ahr *(arylhydrocarbon receptor) and *Zfp277 *(zinc finger protein 277) both contain non-synonymous SNPs. *Zfp277 *also showed differential expression in whole brain and was cis-regulated. Three genes and one predicted gene showed a circadian pattern of expression in liver, including *Zfp277*.

**Conclusion:**

We not only fine-mapped the QTL for circadian period on chromosome 12 but found a new QTL there as well: an association with the timing of the nocturnal activity-minimum. Candidate quantitative trait genes in this QTL are zinc finger protein 277 and arylhydrocarbon receptor. Arylhydrocarbon receptor is structurally related to *Bmal1*, a canonical clock gene.

## Background

Many genes control circadian period in mice [1-5]. Identification of much of the genetic underpinnings of circadian rhythmicity and mechanisms of circadian timekeeping of mice comes from studies using induced mutations, targeted knockout mutations, transgenics, and homologies to *Drosophila *clockwork [6-11]. As insight into the molecular structure of mammalian clocks advanced, the extent of integration of the circadian clockwork with metabolism and the cell cycle was realized [12-14].

Genetic variation in natural populations holds unique clues to gene function. Consequently, identifying and characterizing the molecular machinery of natural variations can open new vistas onto the molecular mechanisms of complex traits like circadian rhythms. Genes that contribute to expression of complex, multi-gene traits are quantitative trait loci (QTL) [15].

The five studies described next suggest the presence of QTL for interstrain differences in circadian period on proximal mouse chromosome 12 (Chr 12). Two studies in panels of recombinant inbred (RI) mice (the B×D RI panel originating from a C57BL/6J × DBA/2J cross and the C×B RI panel from a BALB/cBy × C57BL/6By cross) associated circadian period of wheel-running with provisional QTL [2,3,16]. Three more studies were done on F_2 _populations. In the first, a genomic survey of F_2 _offspring from a C57BL/6J (B6) × BALB/cJ cross, Shimomura (2001) found a QTL (*Frp-3, free-running period 3*) at about 46 megabase pairs (Mbp) [5]. In the second, in an F_2 _from CS × B6 cross Suzuki, 2001 found a QTL for wheel-running period near 80 Mbp [17]. Finally, in an F_2 _intercross of RI mouse strains (BXD19 and CXB07) we found a QTL for circadian period of general locomotor activity near 36 Mbp with a LOD score (a statistical estimate of whether two loci are likely to lie near each other on a chromosome) greater than five. A targeted extension study confirmed *Cplaq10 *(*circadian period of locomotor activity 10*) [18].

Screening B×D RI mice predicted that compared to B6 alleles, DBA/2J (D2) alleles around *Cplaq10 *would produce a short circadian-period phenotype [16]. To test this and to refine the QTL mapping, we compared the phenotypes of B6 and B6.D2NAhrd/J (AhR) strains. AhR mice have a B6 genome except for an insert of D2 DNA spanning roughly 35 to 41 Mbp on Chr 12 [19]. If the AhR mice have a different circadian period than the B6 background strain; then the small D2 insert contains a QTL for the difference in circadian period between B6 and D2 mice.

To gain further information about a possible QTL in this region, we also compared circadian phenotypes of B6 mice with A/J mice and the consomic strain C57BL/6J-Chr 12^A/J^/NaJ (C12A). The C12A strain has an entirely B6 genome except for Chr 12: the homologous Chr 12 from the A/J inbred strain replaces it. If the C12A mice have a different phenotype from B6, then A/J alleles on Chr 12 also associate with it.

## Methods

Mice were purchased from Jackson Laboratories or bred in house. They were acclimated under alternating 200 lux light and dark of 12 hours each (LD 12:12) for at least two weeks prior to the start of the study. Food (Teklad 7001 Mouse & Rat Diet 4%) and water were continuously available throughout the study. The mice assessed were 30 to 150 days old. In Experiment 1 we compared 19 B6 mice and 30 AhR mice. In Experiment 2 we compared 23 B6 mice, nine A/J mice and 33 C12A mice. All animals were maintained in facilities fully accredited by the Association for the Assessment and Accreditation of Laboratory Animal Care. All research protocols and animal care were approved by the Institutional Animal Care and Use Committee in accordance with the guidelines of the Guide for the Care and Use of Laboratory Animals (Institute of Laboratory Animal Resources, Commission on Life Sciences, National Research Council, 1996).

### Experimental housing and care

After acclimation mice were moved into LD 12:12 and housed singly in polycarbonate cages (LXHXW: 12 × 8 × 6 in). Amount of each mouse's activity was acquired by a passive infrared detector mounted above the cage. All test mice were kept in a sound attenuating, ventilated room at a constant temperature (23°C) and humidity. Sound attenuating, opaque dividers were placed between the test cages.

After at least two weeks in LD 12:12, the lights were turned off at the usual time of lights-off to start two weeks of constant darkness (DD). Under DD caretakers wore a Pelican Versabrite headlamp fitted with a red safelight beam diffuser. Care in the darkroom consisted of ten min per day and each mouse was inspected for less than a minute. Daily visits occurred at random times between 8 am and 5 pm.

### Locomotor activity assessment

Daily locomotor activity of the mice was monitored with passive infrared detectors (Ademco, Syosset, NY) mounted over each cage. The passive infrared (ir) proximity sensor works by emitting pulses of ir light, and then measuring the distance to objects from the flight time of the reflected signal. Whenever the distances change, the detector opens or closes a switch. All detectors were tested to ensure response uniformity.

### Calculating timing of nocturnal activity minimum (siesta) under LD 12:12

Prior to assessing the free-running period we assessed the rest-activity patterns under an alternating light and dark condition to determine phase angle of entrainment and placement of the daily activity minimum during that part of LD cycle when the mice were active.

After at least two weeks in LD 12:12 the timing of the siesta and the phase angle of entrainment were calculated using the "Activity Profile" module in Clocklab (Actimetrics Corp, Evanston, IL) a software package for the analysis and display of circadian activity data. Activity Profile plots the average activity for specified dates as a function of circadian time. We grouped activity events into thirty minute bins and calculated the mean activity during the last eight days under LD 12:12. We assessed the phase of minimum activity during the dark part of the cycle.

### Calculating circadian period of locomotor activity

After at least two weeks in DD the circadian period was calculated from the last ten contiguous days of actigraphic records using the *X*^2 ^periodogram analysis in Clocklab. The mean activity was calculated using the "Activity Profile" module.

### Statistical treatment of data

Experiment 1: For activity in LD 12:12, B6 and AhR mice were compared in t-tests for mean activity, phase angle of entrainment, and phase of the siesta. For activity in DD, the two strains were similarly compared for circadian period and mean activity. The effect size of the QTL was calculated from the t-test [20].

Experiment 2: The circadian periods of B6, C12A and A/J mice were compared in a one-way ANOVA with post-hoc Tukey t-test.

### Non-synonymous coding sequence polymorphisms (ncSNP) in the QTL interval

The mouse phenome SNP [21] and the Ensembl Mouse dbSNP 126/Sanger [22] databases were both queried within the QTL interval for the most complete collection of known single nucleotide polymorphisms (SNP) that cause non-synonymous coding sequence variants between the B6 and D2 strains.

### DNA isolation and genotyping microsatellite polymorphisms

DNA isolation and genotyping microsatellite polymorphisms and SNPs were performed by Harlan GenScreen™ (Indianapolis, IN). Mice were genotyped at the following microsatellite and SNP markers (NCBI Build 36.1 Mbp position): D12Mit242 (30.8 Mbp), D12Mit60 (35.4 Mbp), D12Mit153 (35.8 Mbp), rs29213248 (39.3 Mbp), rs29155751 (40.0 Mbp), rs29161407 (40.9 Mbp) and D12Mit2 (42.5 Mbp) [23]. Results of genotyping and characterizing mice for circadian period were combined to identify the Chr 12 region holding the QTL for circadian period.

### Expression Analysis: B6 vs D2

A whole brain database (B6 vs D2) was examined for probe-sets mapping to Chr 12 that were differentially expressed between B6 and D2 mice. Two expression databases were developed in the laboratory of Robert Hitzemann at Oregon Health Sciences University. The development of the databases is described in Hofstetter [24]. Briefly, mice were maintained in LD 12:12 (lights off at 7 pm). Whole brains were taken between 10 AM and 2 PM. The B6 vs D2 database used 6 male mice of each strain. The B6D2F_2 _database used brains from 56 mice (29 females and 27 males). Whole brain RNA was hybridized to the Affymetrix 430A and B arrays. Microarray data was analyzed using the position-dependent nearest neighbor analysis (PDNN; [25,26]). The R program (R Development Core Team, 2005 [27]) was used to calculate the q value [28], which is similar to the well known p value, except that it measures significance in terms of the false discovery rate rather than the false positive rate.

### Expression QTL (eQTL) analysis

The B6D2F_2 _whole-brain database was queried to determine which Chr 12 probe-sets differentially expressed between B6 and D2 had cis- and trans-regulation. The B6D2F_2 _whole-brain database is available online at WebQTL [29]. The analysis of the dataset is described in Hofstetter [24], Hitzemann [30], and Peirce [31]. The computer program HAPPY was used for permutation testing [32]. This was done chromosome by chromosome for all transcripts on the microarray (~45,000); 200 permutations of the data were performed. The 95% threshold for a significant cis-regulated transcript was 4.3 for Chr 12. This was also the average across chromosomes; the difference between chromosomes was ~0.1 LOD units. For trans-regulated transcripts the analysis is genome-wide and thus, the threshold must be increased to 5.7 to account for all 20 chromosomes.

We presumed cis-regulation when a QTL affecting transcript abundance (eQTL) maps near the transcript's chromosomal origin (± 15 cM). We also queried the B6D2F_2 _expression datasets to determine which transcripts showed significant (LOD > 5.7) trans-regulation. These were transcripts originating from genes which were physically located on different chromosomes from the eQTL but mapped with a significant LOD to the QTL. This measure was inherently less precise than the measure of cis-regulation. Consequently, given the limited sample size one can conclude only that the transcript mapped near the QTL.

### Circadian cycling of genes

We examined a database of circadian gene expression [33] to determine if any genes in the QTL had rhythmic gene expression (personal communication: J. Hogenesch, 2007).

## Results

### Ahr vs B6 in DD

**Figure 1:**
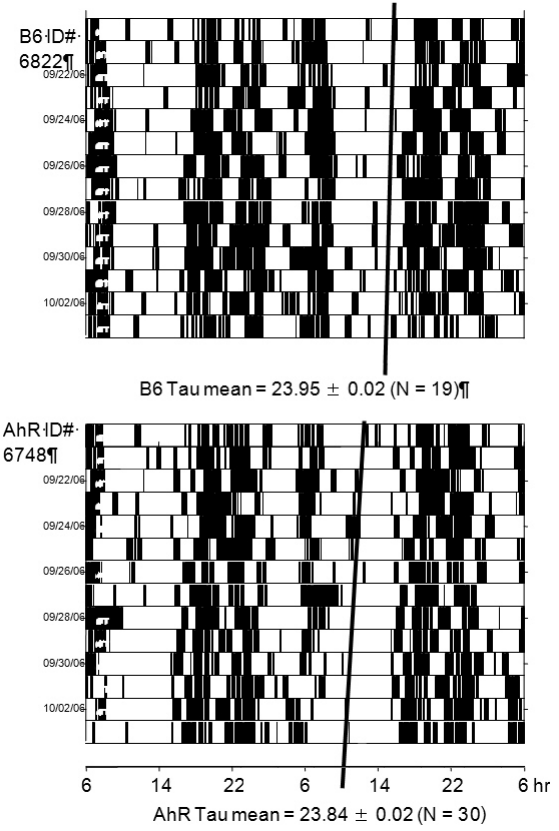
**Raster actograms of locomotor activity of representative mice of the B6 and AhR strains**. Locomotor activity was monitored by infrared motion detectors. Each line of recorded activity is 48 hr. Each pair of days is plotted beneath the previous pair of days. Activity is indicated by the height of the narrow histograms each 10 min wide.

**Figure 2:**
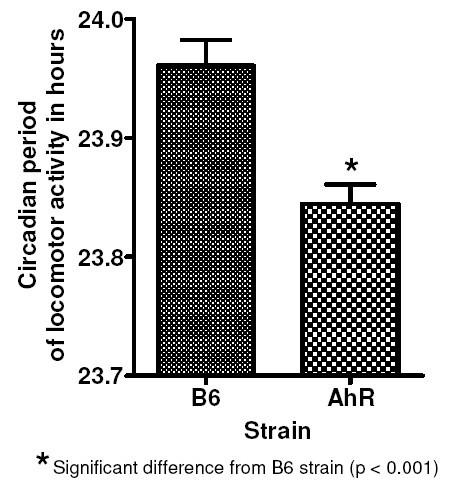
**Circadian period in hours of B6 and AhR strains of inbred mice**. Error bars represent the SEM.

Locomotor activity of representative B6 and AhR mice in DD is shown in Figure 1. There was no difference in the mean locomotor activity between the two strains. The circadian periods of locomotor activity were: B6 mice 23.95 ± 0.02 (SEM) and AhR mice 23.84 ± 0.02 (Figure 2). The strains differed in mean period by t-test (p < .0005). Thus, for the circadian period of locomotor activity, the AhR insert appears to capture the QTL. The effect size of the QTL was 34%.

### AhR vs B6 in LD 12:12

**Figure 3:**
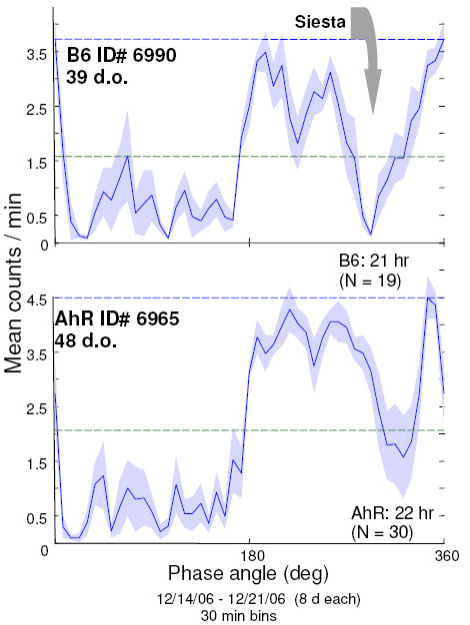
**Daily profile of locomotor activity of representative mice of the B6 and AhR strains in LD 12:12 as a function of circadian time**. Profiles were generated in the "Activity Profile" module in Clocklab (Actimetrics Corp, Evanston, IL) which averaged the activity profiles for the last eight days under LD 12:12. The gray arrow shows the placement of the siesta. The dark line indicates the mean, the shaded areas are SEM.

The patterns of rest/activity in LD 12:12 for representative B6 and AhR mice are shown in Figure 3. The mean phase of the siesta for AhR mice was CT 22.1 ± 0.4 while that of the B6 mice was CT 21.0 ± 0.2. The timing of the siesta under LD 12:12 of the AhR and B6 strains differed by t-test (p < 0.05). Consequently, the AhR insert also contains a QTL for the siesta.

There was no difference between AhR and B6 for mean activity or phase angle of entrainment in LD 12:12. Consequently, the AhR insert does not appear to contain QTL for these traits.

### B6, C12A, and A/J in DD

**Figure 4:**
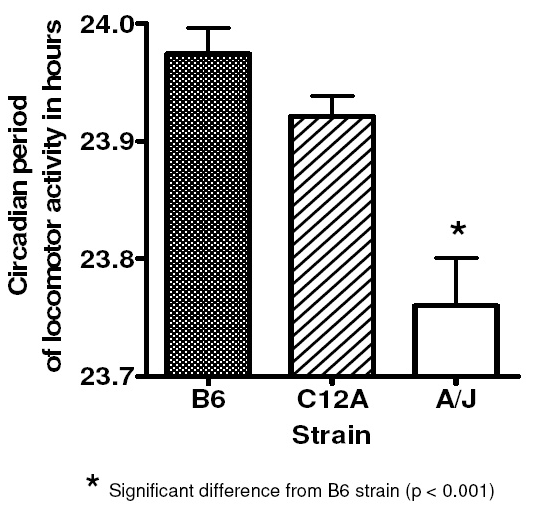
**Circadian period in hours of B6, C12A, and A/J strains of inbred mice**. Error bars represent the SEM.

The circadian periods of locomotor activity were: B6 mice 23.97 ± 0.02, A/J mice 23.76 ± 0.04, and C12A mice 23.92 ± 0.02 (Figure 4). There was a significant effect of strain by one-way ANOVA [F(2,62) = 3.14, p < .0001]. Although B6 and A/J strains differed in mean period by post-hoc t-test (p < .001), there was no difference between B6 and C12A strains. Thus, A/J alleles on Chr 12 do not appear to contribute to the period difference between B6 and A/J.

### Definition of the QTL interval

The results of genotyping are in Table 1. The D2 insert in AhR mice extends from D12Mit60 at 35.4 Mbp to rs29161407 at 41.0 Mbp. The QTL spans 5.6 Mbp (NCBI Build 36.1 Mbp positions). This genomic insert contains 15 genes, and 12 predicted genes.

**Table 1:** Phenotype and genotype of AhR and B6 mice

		**AhR**	**B6**
**Circadian period (h)**		23.83 ± 0.02	23.96 ± 0.02
**Marker**	**Mbp**		
D12Mit242	30.8	B6:B6	B6:B6
D12Mit60	35.4	***D2:D2***	B6:B6
D12Mit153	35.8	***D2:D2***	B6:B6
rs29213248	39.3	***D2:D2***	B6:B6
rs29155751	40.0	***D2:D2***	B6:B6
rs29161407	40.9	***D2:D2***	B6:B6
D12Mit2	42.5	B6:B6	B6:B6

B6:B6 – homozygous for C57BL/6J alleles

**D2:D2 **– homozygous for DBA/2J alleles

Mbp – position from NCBI Build 36.1

### ncSNP in the QTL interval

Candidate genes within the QTL are shown in Table 2. Three genes in the 5.6 Mbp QTL interval contain ncSNPs between B6 and D2 strains; *Ahr *(arylhydrocarbon receptor), *Meox2 *(mesenchyme homeobox 2) and *Zfp277 *(zinc finger protein 277).

**Table 2:** Candidate QTG in the *Cplaq10 *genomic region

**Gene**	**Start (Mbp)**	**Diff expr**	**Cis-reg**	**ncSNP**	**Circ cyc**	**Ensembl description**
*Ahr*	36.08	No		6 SNP	No	aryl-hydrocarbon receptor
*Meox2*	37.62	No		1 SNP	No	mesenchyme homeobox 2
*Arl41*	40.54	No		No	Yes	ADP-ribosylation factor-like 4A
*A530016O06Rik*	37.75	No		No	Yes	RIKEN cDNA A530016O06 gene
*Ifrd1*	40.71	No		No	Yes	interferon-related developmental regulator 1
*Zpf277*	40.83	Yes	Yes	2 SNP	Yes	zinc finger protein 277

Start (Mbp): from NCBI Build 36.1

Diff expr: Differential expression in whole brain B6 vs D2

Cis-reg: Cis-regulated

ncSNP: non-synonymous coding SNP

Circ cyc: Circadian cycling in liver

### Expression analysis

The expression analysis of whole brain had 29 probe-sets representing 14 of 15 known genes in the QTL, and 12 probe-sets representing 9 of the 12 predicted genes. In total, the chips had probe-sets for 85% of the genes in the AhR insert. The only gene in the QTL differentially expressed between B6 and D2 strains was *Zfp277*; it was also cis-regulated. The eQTL analysis suggested that the QTL region of Chr 12 might control by trans-regulation expression of two genes on Chr 6 (1700019G17Rik and ribosomal protein S2) and two unknown genes, one on distal Chr 12 and one in an unknown location of the mouse genome.

### Circadian cycling of genes

Twenty of the genes and predicted genes in the QTL were represented in the circadian gene expression database. Three genes and one predicted gene showed circadian cycling in mouse liver but not pituitary (q < .01). The predicted gene was RIKEN cDNA A530016O06, and the three genes were *Arl41 *(ADP-ribosylation factor-like 4A), *Ifrd1 *(interferon-related developmental regulator 1), and *Zfp277*.

## Discussion

Short circadian period in the AhR congenic mice relative to their B6 progenitors confirms that the AhR insert on proximal Chr 12 contains a QTL for circadian period, designated *Cplaq10*. It extends from 35.4 to 41.0 Mbp and accounts for 34% of the total phenotypic variance in period, a large effect. Reasonable effect size makes it more likely that we will be able to identify the responsible quantitative trait genes (QTG) [34].

Since B6 and C12A mice showed no difference in period, the most parsimonious explanation is that genes on Chr 12 do not contribute to the difference between B6 and A/J. A less likely explanation is that the phenotypic difference arises from multiple QTL on Chr 12: one set increases period; the other decreases it; the net effect is zero.

Traditionally the period of wheel-running is the preferred phenotype in circadian rhythms research. However, wheel-running both alters the circadian timekeeping of mice and adds considerable environmental variance to it [35]. Edgar et al (1991) showed that access to a running-wheel shortened the period of mice [36]. When we mapped each phenotype (period of wheel-running and general locomotor activity) in BXD RI mice we found few to no overlapping QTL; they were influenced by different genes [3,16]. Moreover, when we mapped both phenotypes in the same group of F_2 _mice, we found two genome-wide associations for general locomotor activity but none for wheel-running [18]. Therefore, period of general activity has a larger effect size than period of wheel-running [34]. For mapping QTG of circadian rhythms, we concluded that calculating period from locomotor activity was a better choice than from wheel-running.

In the timing of the siesta (a feature common to the locomotor activity profile of certain strains of inbred mice) B6 and AhR differed. About 8–9 hours after their activity begins the B6 strain has a characteristic siesta; in AhR it is an hour later. For timing of the siesta, the AhR insert on Chr 12 captures its QTL. Perhaps, in the interaction between the arousal state and the circadian activity cycle, B6 and Ahr differ as well.

The AhR insert contains fifteen genes and twelve predicted genes. To identify candidate QTGs, we screened the resident genes for the following: non-synonymous coding SNPs: gene expression differences between B6 and D2 in whole brain; cis- and trans-regulation of expression; and circadian gene expression. Several studies integrate behavioral QTL and genome-wide gene expression data to identify candidate QTGs [24,37-43].

A candidate gene in this area is zinc finger protein 277 (*Zfp277*); it stands out because all the following criteria were met: it contains ncSNPs; it shows differential expression in whole brain; it is cis-regulated; and it shows circadian cycling of expression. Apparently, zinc finger proteins can modulate the circadian clock. The promoter region of *mPer1 *contains targets for zinc finger protein binding and is essential in NG108–15 cells for CaM kinase II-induced gene-activation [44]. Furthermore, mouse LARK protein not only contains a zinc finger element but also modulates post transcriptional expression of *mPer1*; however, in this case, the element may not activate *mPer1 *[45].

Another interesting candidate, *Ahr*, codes for arylhydrocarbon receptor (Ahr). As a member of a transcription-factor family related structurally to *Bmal1 *(a canonical clock gene), it contains the following: a basic helix-loop-helix-periodicity/arylhydrocarbon nuclear transporter/simple-minded (bHLH/Per-Arnt-Sim) motif and six ncSNPs. Also known as the dioxin receptor (a ligand-activated transcription factor), it is expressed in brain. Although its physiologic role is not known, it is highly conserved evolutionarily. There is evidence that it regulates light-influenced circadian behaviors [46].

Suggestive of additional support that *Ahr *is a QTG are the following: B6 alleles (*Ahr*^*b*-1^) with both high ligand-affinity (K_D _= 0.65 nM) and high receptor concentration (B_max _= 151 fmol/mg protein); and D2 alleles (*Ahr*^*d*^) with low (10-fold less than B6) [47]. Unfortunately, a preliminary report finds that circadian period of wheel-running does not differ between *Ahr *knock-out and B6 strains [46].

Although SNP typing of A/J mice supports *Zfp277 *as the candidate QTG, it does not support *Ahr*. If a SNP is responsible for the difference in period, we expect B6 alleles to associate with long period and D2 alleles to associate with short period. Since C12A mice have long period like B6, we expect them to have the same alleles as B6 at the critical SNP. This is true in *Zfp277*: at both of the ncSNPs where B6 and D2 differ, C12A mice have the same allele as B6. However, at five of the six ncSNPs in *Ahr *where B6 and D2 differ, C12A mice have the same allele as the strain with the short period, D2.

There are a number of caveats to put forward when integrating QTL and gene expression data. Within any interval the Affymetrix array surveys some of the known and predicted genes. Representation is 85% for our interval, so there may not be an Affymetrix probe-set for the true QTG. Furthermore, some gene products have multiple probe-sets, but only one probe-set may show differential expression. Consequently, we may have failed to detect differential expression of a particular transcript. This is especially true for genes with only a single probe-set. There were several trans-regulated transcripts that mapped to the interval of interest but we were unable to link any transcription factor or factors within the interval to trans-regulated genes. The complexity of the relevant biology makes these analyses preliminary at best. Finally, we used whole-brain datasets taken at a single circadian time. Differential expression of several genes in the QTL might be found by examining tissue from the suprachiasmatic nucleus (SCN) taken at several times across the circadian cycle.

The current study is an example of using multiple resources and strategies to characterize a QTL. In future work we will assess the period of generalized locomotor activity of *Ahr *knock-out mice compared to their background strain. It is possible that the period of generalized locomotor activity differs from the period of wheel-running. We will also perform real-time quantitative PCR of candidate QTG using SCN tissue. If these steps support a candidate QTG, we will use BAC gene transfer to confirm them [48].

## Competing interests

The author(s) declare that they have no competing interests.

## Authors' contributions

JRH conceived of the C12A study, was responsible for the design and coordination of the entire study, and helped draft the manuscript. DS developed the protocol for characterizing siesta, and drafted the manuscript. AM conceived of the AhR study and performed the statistical analyses. All authors edited and approved the final manuscript.
